# Validation of the children international IgA nephropathy prediction tool based on data in Southwest China

**DOI:** 10.3389/fped.2023.1183562

**Published:** 2023-06-23

**Authors:** Xixi Yu, Jiacheng Li, Chengrong Tao, Jia Jiao, Junli Wan, Cheng Zhong, Qin Yang, Yongqi Shi, Gaofu Zhang, Haiping Yang, Qiu Li, Mo Wang

**Affiliations:** ^1^ Department of Nephrology, Ministry of Education Key Laboratory of Child Development and Disorders, National Clinical Research Center for Child Health and Disorders, China International Science and Technology Cooperation Base of Child Development and Critical Disorders, Children’s Hospital of Chongqing Medical University, Chongqing, China; ^2^ Department of Hematology and Oncology, Children's Hospital of Chongqing Medical University, Chongqing, China

**Keywords:** IgA nephropathy, prediction tool, validation, chronic kidney disease, pediatric

## Abstract

**Background:**

Immunoglobulin A nephropathy (IgAN) is one of the most common kidney diseases leading to renal injury. Of pediatric cases, 25%–30% progress into end-stage kidney disease (ESKD) in 20–25 years. Therefore, predicting and intervening in IgAN at an early stage is crucial. The purpose of this study was to validate the availability of an international predictive tool for childhood IgAN in a cohort of children with IgAN treated at a regional medical centre.

**Methods:**

An external validation cohort of children with IgAN from medical centers in Southwest China was formed to validate the predictive performance of the two full models with and without race differences by comparing four measures: area under the curve (AUC), the regression coefficient of linear prediction (PI), survival analysis curves for different risk groups, and R_2_D.

**Results:**

A total of 210 Chinese children, including 129 males, with an overall mean age of 9.43 ± 2.71 years, were incorporated from this regional medical center. In total, 11.43% (24/210) of patients achieved an outcome with a GFR decrease of more than 30% or reached ESKD. The AUC of the full model with race was 0.685 (95% *CI*: 0.570–0.800) and the AUC of the full model without race was 0.640 (95% *CI*: 0.517–0.764). The PI of the full model with race and without race was 0.816 (*SE* = 0.006, *P *< 0.001) and 0.751 (*SE* = 0.005, *P *< 0.001), respectively. The results of the survival curve analysis suggested the two models could not well distinguish between the low-risk and high-risk groups (*P *= 0.359 and *P *= 0.452), respectively, no matter the race difference. The evaluation of model fit for the full model with race was 66.5% and without race was 56.2%.

**Conclusions:**

The international IgAN prediction tool has risk factors chosen based on adult data, and the validation cohort did not fully align with the derivation cohort in terms of demographic characteristics, clinical baseline levels, and pathological presentation, so the tool may not be highly applicable to children. We need to build IgAN prediction models that are more applicable to Chinese children based on their particular data.

## Introduction

Immunoglobulin A nephropathy (IgAN) is the most common type of primary glomerulonephritis in the world (1, 2), accounting for more than 50% of primary glomerulonephritis in the Chinese population and approximately 17% of primary glomerulonephritis in Chinese children (3, 4). It is also one of the essential etiologies leading to a decrease in renal function. The long-term prognosis of IgAN is poor, as the majority of Chinses children with IgAN have progressive disease, with 25%–30% of cases reaching end-stage kidney disease (ESKD) within 20–25 years after diagnosis (5). A Japanese study showed that 50% of patients with IgAN developed ESKD over 30 years of follow-up despite treatment (6). Therefore, it is vital to explore the relevant factors affecting the progression of the disease and to provide timely intervention to slow down its progression. Currently, the main clinically significant risk factors, based on adult data, are proteinuria at the time of renal biopsy, hypertension, and reduced glomerular filtration rate (7). The Oxford classification and further observations showed that not only mesangial hypercellularity (M) but also endocapillary hypercellularity (E), segmental glomerular sclerosis (S), tubular atrophy, interstitial fibrosis (T), and crescents (C) are risk factors associated with IgAN (8).

The predictive power of individual risk factors is limited. We need a comprehensive predictive model based on clinical presentation and pathological features to assess the prognosis of the IgAN. There are two predictive models for adults, one based on Chinese patients’ data (9) and the other based on multicentre, multiethnic data (10). One international team has built a predictive model for children by incorporating data from 1,060 pediatric patients and using secondary outcome indicators as endpoint events to adjust the model coefficients (10, 11). Once a model has been constructed, it requires not only internal validation but also external validation to demonstrate generalizability and transportability. Our study aims to validate the above model using data from a medical center in Southwest China.

## Methods

### Patients

**Figure 1:**
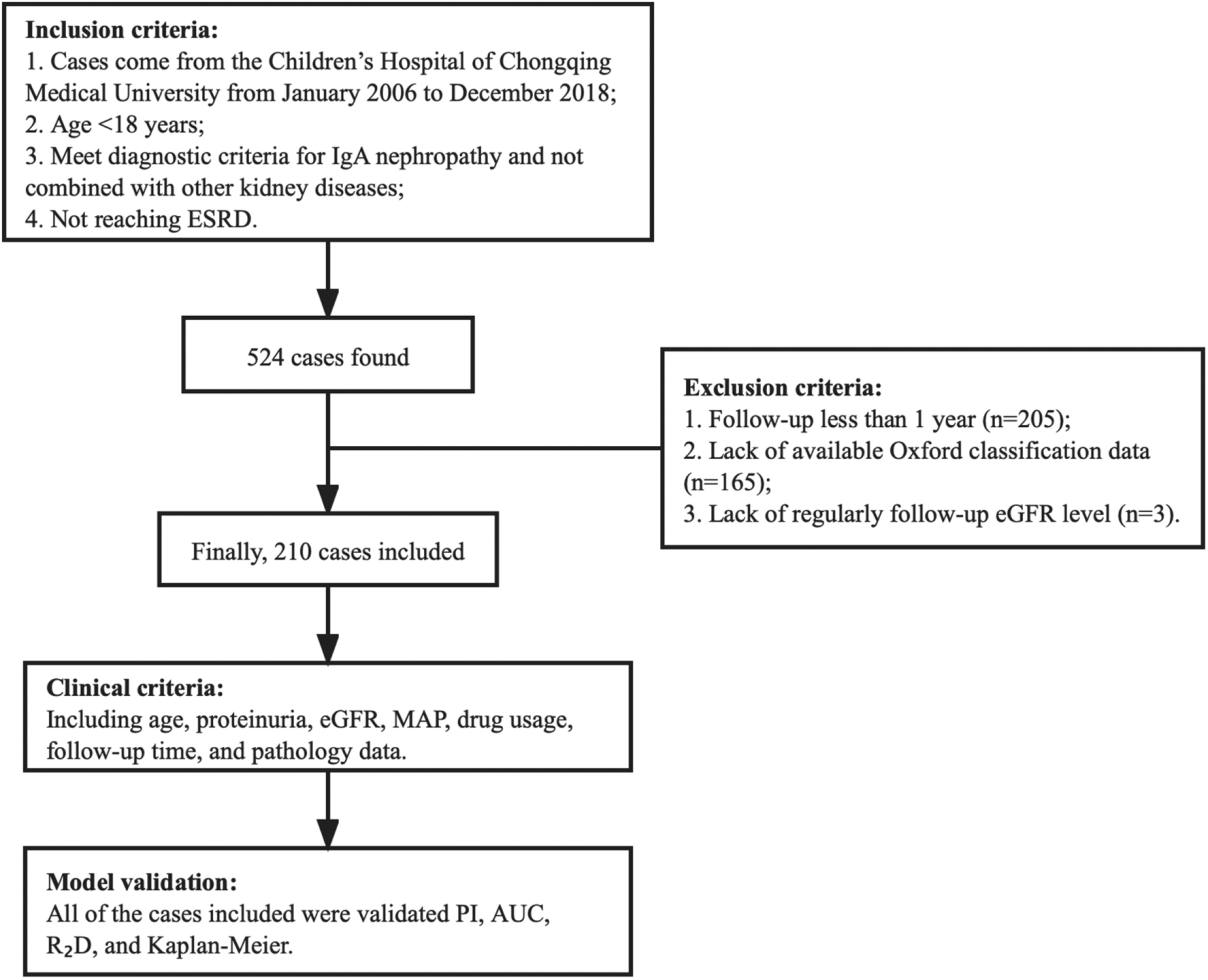
Flow chart of patients finally included in the analysis.

In this study, data from patients who satisfied the following conditions were included: age <18 years, meeting the diagnostic criteria for IgAN, no other kidney diseases, and not reaching ESKD. The exclusion criteria were: a follow-up duration of less than 1 year, a lack of available Oxford classification data, or a lack of regular follow-up estimated glomerular filtration rate (eGFR) levels. We included a total of 524 pediatric patients who were diagnosed with IgAN on renal biopsy and were treated at the Children’s Hospital of Chongqing Medical University from January 2006 to December 2018. All of our patients were Chinese, of whom 210 cases had an available eGFR during follow-up. The flow chart of the patients is shown in Figure 1.

Our cases met the inclusion criteria of the original article (11).

### Definitions

Age, proteinuria, estimated glomerular filtration rate (eGFR was calculated using the Schwartz formula (12), systolic blood pressure (SBP), diastolic blood pressure (DBP), mean arterial blood pressure (MAP = 1/3*SBP + 2/3*DBP), prior use of drugs blocking angiotensin system blockers (RASB, including angiotensin-converting enzyme inhibitors and angiotensin in receptor blockers), at biopsy and at the follow-up to determine the use of immunosuppression, the Oxford classification (the definition of Oxford classification show in Supplementary S3) at biopsy (13). Each covariate is defined precisely according to the original publication of Barbour et al. (11). The primary outcome was the first occurrence of either ESKD (eGFR < 15 ml/min/1.73 m^2^, dialysis or transplantation) or a reduction in eGFR below 50% of the value at biopsy. The secondary outcome was defined similarly using a 30% reduction in eGFR or ESKD.

### Prediction models for external validation

The prediction models for external validation were derived from the original publication of Barbour et al. The prediction models in the report were obtained by updating formula coefficients and using secondary outcome indicators as endpoint events from a clinical prognostic model for adult patients with IgAN constructed by the same team. They included age at renal biopsy, MAP at renal biopsy, eGFR levels at renal biopsy, Oxford classification, and the use of RASB and immunosuppression at renal biopsy as variables for model construction. The model was divided into two models with or without the inclusion of race differences, the full model with race and the full model without race.

### Statistical analysis

The age changes of the verified patients were normally distributed and usually described as mean and standard deviation, but the derivation cohort was described as median and quartiles, which were chosen to maintain consistency between them. The remaining indicators, such as follow-up time, eGFR, proteinuria, and MAP, were described using median and quartiles.

To verify the model, we first calculated the linear predictive factor of each patient in the current cohort based on the paper entitled *Updating the international IgA nephropathy prediction tool for use in children* (11), the exact predictive factors, and coefficient values. Details of the formula can be found in Barbour SJ et al. The address for the online formula is https://qxmd.com/calculate/calculator_713/international-igan-prediction-tool-at-biopsy-pediatrics#.

#### Linear prediction coefficients

The regression coefficient of the linear prediction coefficients of the two models were estimated to evaluate the discrimination. We used an interval format Cox proportional hazards model for both models (14). A linear predictor >1 indicates the model is acceptably distinguished, and vice versa.

#### Receiver operating characteristic curve

The receiver operating charateristic curve (ROC) and area under the curve (AUC) were used to determine the ability to discriminate in the full model with race and the full model without race. By definition, the AUC must be between 0.5–1, and the AUC for acceptable discriminatory ability is generally ≥0.65 (15). In addition, the coefficient of determination (R_2_D) was used to assess the goodness of fit.

#### Kaplan-Meier curve

We grouped the risks calculated from the linear predictor formula for each patient according to <16th centile (low-risk group) and approximately from the 16th to <50th centile (medium-risk group), the 50th to <84th centile (high-risk group), and >84th centile (extremely high-risk group) (14). Subgroup analyses were performed to derive survival curves. All data were analyzed using SPSS 26.0 statistical software and the R Programming Language.

## Results

### Clinical characteristics, treatment, and outcome of baseline patients

In total, 524 pediatric patients hospitalized at the Children’s Hospital of Chongqing Medical University, the National Children’s Regional Medical Center, were involved in this study, mainly from 36 municipal districts of four provinces in Southwest China (Sichuan, Guizhou, Chongqing, and Yunnan provinces).

All patients’ clinical characteristics are shown in Table 1. A total of 210 patients were included in our cohort, all of whom were Chinese. The mean age of the patients was 9.43 ± 2.71 years, and 129 were males. The median level of eGFR at the time of renal biopsy was 155.63 ml/min/1.73 m^2^. Most children were in CKD stage 1 (90.95%), and only 1.43% were in CKD stage 4. The median proteinuria at renal biopsy was 0.97 g/day/1.73 m^2^ (IQR: 0.39–1.80 g/day/1.73 m^2^), with 51.43% of children having less than 1 g/day/1.73 m^2^, 38.10% having between 1 and 3 g/day/1.73 m^2^, and only 10.48% having more than 3 g/day/1.73 m^2^. The median MAP at renal biopsy was 82.00 mmHg.

**Table 1:** Comparison of clinical and histological characteristics in the current and previously reported cohorts.

Characteristics	Reported derivation cohort	This validation cohort	*P*
Number of patients	1,060	210	
Follow-up (years)	3.9 [2.1,6.4]	2.58 [1.48,3.69]	
Age (years)	12.7 [9.6,15.4]	9.29 [7.48,11.25]	
Male sex	687 (64.8%)	129 (61.43%)	
Race			
Caucasian	326 (31%)		
Chinese	221 (21%)	210 (100%)	<0.001*
Japanese	422 (40.1%)		
Other	83 (7.9%)		
eGFR at biopsy (ml/min/1.73 m^2^)	98 [79,118]	155.63 [119.66,189.70]	
<30	14 (1.4%)	3 (1.43%)	0.901
30–60	78 (7.4%)	7 (3.33%)	0.033
60–90	318 (30%)	9 (4.29%)	<0.001*
>90	650 (61.3%)	191 (90.95%)	<0.001*
Standardized MAP (mmHg)	85.1 [77.3,92.9]	82.0 [76.67,90.42]	
Proteinuria (g/day/1.73 m^2^)	1.2 [0.5,3.0]	0.97 [0.39,1.80]	
<0.5	266 (26.3%)	65 (30.95%)	0.077
0.5–1	180 (17.8%)	43 (20.48%)	0.224
1–2	201 (19.9%)	56 (26.67%)	0.011
2–3	115 (11.4%)	24 (11.43%)	0.808
>3	250 (24.7%)	22 (10.48%)	<0.001*
Histopathology			
M1	550 (52%)	203 (96.67%)	<0.001*
E1	416 (39.2%)	105 (50.0%)	0.004
S1	541 (51%)	31 (14.76%)	<0.001*
T1	146 (13.8%)	23 (10.95%)	0.271
T2	11 (1%)	1 (0.48%)	0.442
Primary outcome			
50% decline in eGFR	42 (4%)	6 (2.86%)	0.443
ESKD	33 (3.1%)	4 (1.90%)	0.341
Total primary outcome	52 (4.9%)	10 (4.76%)	0.930
Secondary outcome:			
30% decline in eGFR	112 (10.6%)	24 (11.43%)	0.712

^*^ Indicates that the data is statistically different between the two cohort.

In the renal biopsy, M1 occurred in the majority of children (96.67%), whereas E1 was present in half of the total (50%). In contrast, children presenting S1, T1, and T2 were rare, accounting for 14.76%, 10.95%, and 0.48% of the total, respectively.

Our medical center standardizes the treatment of patients according to the guidelines for the diagnosis and treatment of primary IgA nephropathy published by the Chinese Society of Pediatric Nephrology Medical in 2017 (16). Steroids combined with immunosuppressive therapy were given to children with nephrotic proteinuria, moderate to severe mesangial proliferation, or crescent formation. The immunosuppressant cyclophosphamide is the first-line choice, but mycophenolate mofetil alone is used in approximately 4.96% of the children in our medical center. These patients’ renal biopsies did not show significant tubular atrophy or interstitial fibrosis, and their 24-hour proteinuria quantification was less than 50 mg/kg at the time of presentation. Most of the children were taking angiotensin-converting enzyme inhibitor (ACEI) drugs, mainly captopril, to reduce proteinuria. The kidney disease: improving global outcomes (KDIGO) guidelines have no specific recommendations for the use of steroids or immunosuppressants in pediatric patients (17).

The median follow-up time in this cohort was 2.58 years (IQR:1.48–3.69), and the percentage of children achieving the primary outcome indicator was 4.76%, with 2.86% achieving an eGFR decline of more than 50% and 1.90% developing ESKD. In total, 11.43% of children were seen if the secondary outcome indicator was used as the endpoint event.

### Regression on linear predictor in validation data

The calibration slopes of the linear prediction were 0.816 (*SE* = 0.006, *P *< 0.001) for the full model with race and 0.751 (*SE* = 0.005, *P *< 0.001) for the full model without race. Thus, discrimination appeared not to be preserved.

### Measures of discrimination and model fit

**Figure 2:**
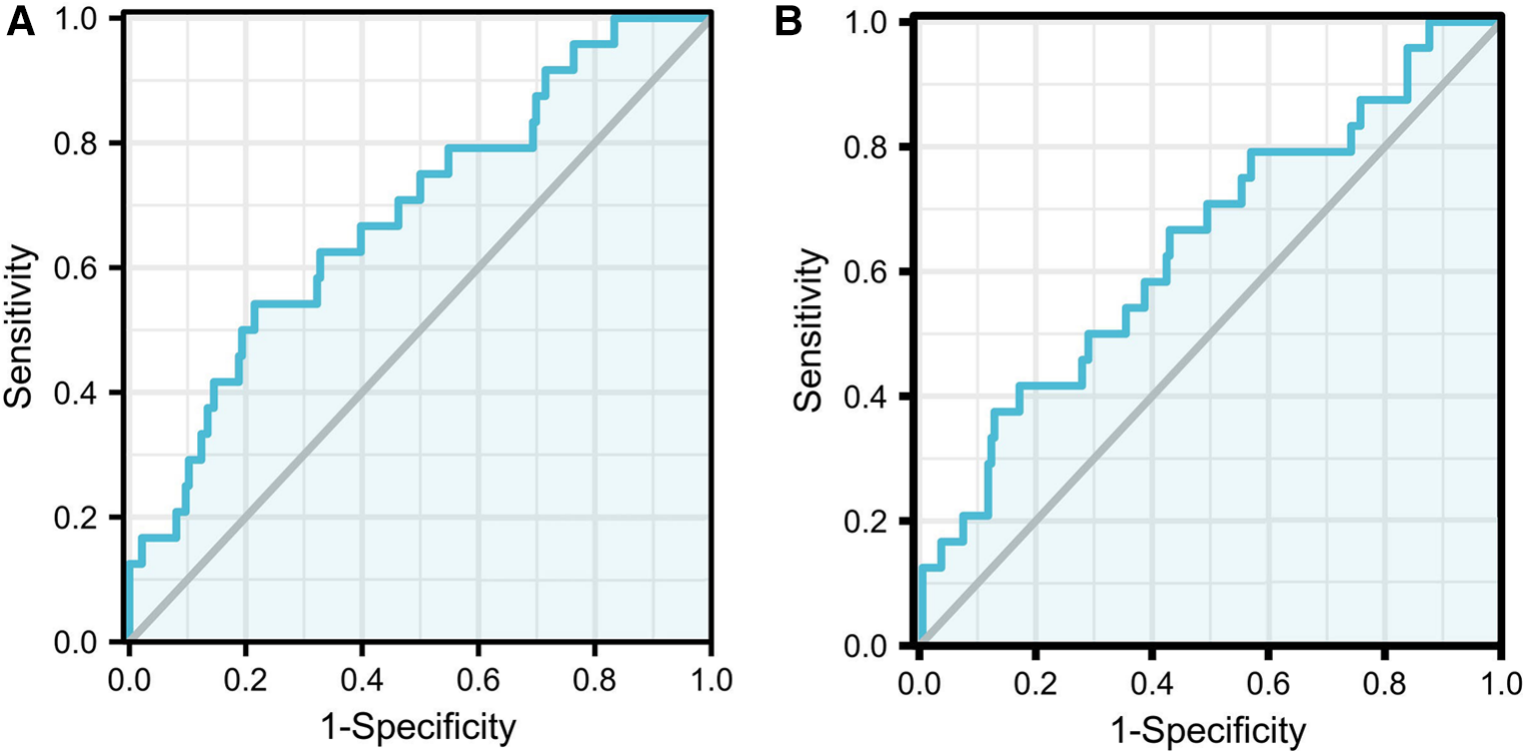
Images of ROC curve. The full model with race AUC:0.685 (95% CI:0.570−0.800 *P* = 0.003) (**A**); The full model without race AUC:0.640 (95% CI:0.517−0.764 *P* = 0.025) (**B**).

By applying the reported models to our current cohort, the C-statistic was calculated to be 0.685 (95%*CI*:0.570–0.800 *P *= 0.003) for the model with race and 0.640 (95%*CI*:0.517–0.764 *P *= 0.025) for the model without race. The details are described in Figure 2.

In addition, R_2_D values were 66.5% for the full model with race and 56.2% for the full model without race. The higher R_2_D values compared to those reported in the derivation cohort were not consistent with linear predictors, and the cloud indicated the good performance of the model fit.

### Comparison of risk groups

**Figure 3:**
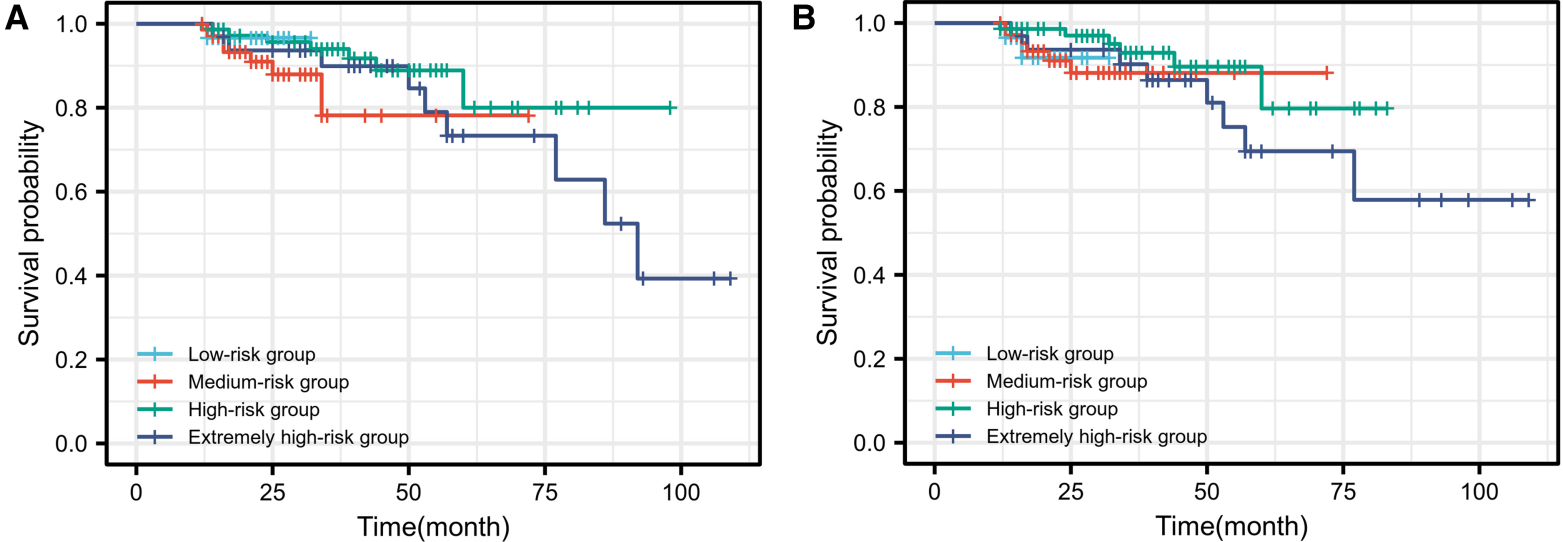
Kaplan-Meier curves for survival probability of the primary outcome in four risk groups based on the percentile of the linear predictor. The full model with race (**A**). The full model without race (**B**). Blue denotes the low-risk group, red is the medium-risk group, green is the high-risk group, and navy is the extremely high-risk group.

The clinical and pathological characteristics of the patients based on the full model with race and the full model without race grouping are shown in Table 2. We found that in terms of clinical presentation, the higher the risk grouping, the longer the follow-up, the older the age at the time of renal biopsy, and the higher MAP at baseline levels, while the better of the level of eGFR showed the opposite presentation to the risk group. From a pathological point of view, the higher the risk grouping, the higher the proportion of S1 and T1 present. Figure 3 shows the result of the Kaplan-Meier curves in four groups based on the percentages of linear prediction.

**Table 2:** Clinical and histological characteristics of groups of patients according to risk stratification based on the full model with (Table 2-1) and without race (Table 2-2).^a^

Table 2-1 The full model with race					
Full model with race	Group 1 (Lower 16th centile) *n* = 32	Group 2 (16–50th centile) *n* = 73	Group 3 (50–84th centile) *n* = 73	Group 4 (Upper 16th centile) *n* = 32	*P*
Follow up (years)	1.42 [1.17,1.90]	1.83 [1.25,2.75]	3.0 [2.38,4.0]	4.04 [2.85,5.0]	<0.001***
Age (years)	7.46 [6.42,9.98]	9.33 [7.63,10.71]	10.25 [8.0,11.92]	10.33 [8.02,12.23]	<0.001***
Male sex	19 (59.4%)	45 (61.6%)	45 (61.6%)	20 (62.5%)	0.994
eGFR at biopsy (ml/min/1.73 m^2^)	94.67 [63.85,121.94]	152.38 [128.09,179.23]	171.86 [129.35,206.76]	180.75 [145.66,245.94]	<0.001***
Standardized MAP (mmHg)	82.67 [72.83,85.25]	80.33 [76.67,86.33]	81.33 [76.67,90.83]	90.0 [79.33,102.0]	<0.05*
Proteinuria (g/day/1.73 m^2^)	1.34 [0.66,2.32]	1.09 [0.36,1.60]	0.76 [0.30,1.78]	0.78 [0.44,1.99]	0.353
Histopathology					
M1	30 (93.75%)	70 (95.5%)	72 (98.6%)	31 (96.9%)	0.488
E1	20 (62.5%)	35 (47.9%)	33 (45.2%)	17 (53.1%)	0.399
S1	0 (0%)	4 (5.5%)	9 (12.3%)	18 (56.3%)	<0.001***
T1	0 (0%)	0 (0%)	6 (8.2%)	17 (53.1%)	<0.001***
T2	0 (0%)	0 (0%)	0 (0%)	1 (3.1%)	0.305
Table 2-2 The full model without race					
Full model without race	Group 1 (Lower 16th centile) *n* = 32	Group 2 (16–50th centile) *n* = 73	Group 3 (50–84th centile) *n* = 73	Group 4 (Upper 16th centile) *n* = 32	*P*
Follow up (years)	1.33 [1.17,1.83]	1.83 [1.25,2.67]	3.33 [2.54,4.33]	4.04 [3.06,5.0]	<0.001***
Age (years)	7.67 [6.44,10.25]	9.33 [7.83,10.88]	9.33 [7.63,11.42]	10.71 [9.0,12.65]	<0.01**
Male sex	18 (56.3%)	44 (60.3%)	46 (63.0%)	21 (65.6%)	0.865
eGFR at biopsy (ml/min/1.73 m^2^)	113.20 [85.22,141.08]	153.34 [125.72,181.55]	169.79 [128.23,204.99]	180.54 [145.66,231.51]	<0.001***
Standardized MAP (mmHg)	76.50 [72.42,84.17]	80.0 [76.67,85.5]	82.67 [77.67,89.83]	91.83 [83.33,103.17]	<0.001***
Proteinuria (g/day/1.73 m^2^)	1.47 [0.06,3.53]	0.96 [0.42,1.62]	0.76 [0.29,1.69]	1.09 [0.52,2.05]	0.227
Histopathology					
M1	29 (90.6%)	71 (97.3%)	71 (97.3%)	32 (100%)	0.211
E1	19 (59.4%)	34 (46.4%)	34 (46.4%)	18 (56.3%)	0.537
S1	0 (0%)	5 (6.8%)	11 (15.1%)	15 (46.9%)	<0.001***
T1	0 (0%)	5 (6.8%)	6 (8.2%)	12 (37.5%)	<0.001***
T2	0 (0%)	0 (0%)	0 (0%)	1 (3.1%)	

^a^ Continuous variables were tested non-parametrically using the ANOVA test, categorical variables were tested using the chi-square test, and statistical significance was considered when *P* < 0.05.

^*^ Indicates *P* value less than 0.05;

^**^ Indicates *P* value less than 0.01;

^***^ Indicates *P* value less than 0.001.

In terms of images, neither model did well at distinguishing patients at different risk levels up to 60 months of follow-up. This can be explained by the inconsistency in the follow-up time of patients in each group. The full model without race better distinguished between the medium-risk group, high-risk group, and extremely high-risk group at the follow-up times beyond 60 months; the low-risk group could not be evaluated due to missing data. After 60 months of follow-up, there was a higher kidney survival rate in the high-risk group than in the medium-risk group in the full model with race.

Both models failed to distinguish between different risk groups through imaging, and this was confirmed by the statistics. The survival curve was validated by the log-rank test, where the full model with race yielded *P *= 0.359, whereas the full model without race yielded *P *= 0.452.

### Model calibration

The protocol is based on the accepted validation method proposed by Royston et al. The calibration of the Cox model can be evaluated by estimating the regression coefficients of the prognostic index(PI) in the cohort (14). First, the PI was calculated for each individual in our cohort according to the formula in the original report. Second, estimates of the regression coefficients for the PI in the validation cohort were calculated. In our data, the calculated estimates of PI for the full model with and without race were 0.816 (*SE* = 0.006 *P *< 0.001) and 0.751 (*SE* = 0.005 *P *< 0.001), respectively, both less than 1. Therefore, neither model appears to show sufficient calibration in this validation cohort to predict the 5 years risk of an endpoint event accurately. This is also reflected in the observed survival rates shown in the Kaplan-Meier curve (Figure 3).

## Discussion

The full model with race and the full model without race reported in this study provide predictive tools for clinical progression in children with IgAN. We enrolled an external cohort using current data from our regional medical centre to further evaluate these two models. For both models with or without race, it was observed that neither had a good discriminatory ability (full model with race: AUC = 0.685 95% CI:0.570–0.800 *P *= 0.003; full model without race: AUC = 0.640 95% CI:0.517–0.764 *P *= 0.025), but they performed better than they did in the original cohort in terms of model fit (full model with race: R_2_D = 66.5%; full model without race: R_2_D = 56.2%). The survival curves for children stratified by the current predictor percentile were not well separated in either model. Our external validation demonstrates that the international IgA nephropathy prediction tool cannot achieve an acceptable level of generalizability and transportability in terms of either discrimination or calibration.

The external validation consisted of only Chinese patients, while the derivation cohort had four ethnicities and was 21% Chinese. Regarding demographic characteristics, children in the initially reported derivation cohort had a longer age at renal biopsy and a longer follow-up than our cohort. In the reported derivation cohort, the median eGFR level at renal biopsy was 98 ml/min/1.73 m^2^ (IQR:79–118 ml/min/1.73 m^2^) compared to 155.63 ml/min/1.73 m^2^ (IQR:119.66–189.70 ml/min/1.73 m^2^) in our cohort. There were 250 children (24.7%) with proteinuria over 3 g/day/1.73 m^2^ in the derivation cohort, but only 22 (10.48%) in our validation cohort. From these two pieces of clinical data, which are closely related to prognosis, it appears that the children in our cohort were somewhat better off at baseline than the derivation cohort. In terms of pathological findings, there were more children with M1 in our validation cohort (96.67% in our validation cohort vs. 52% in the derivation cohort). Additionally, more children had S1 in the originally reported derivation cohort (51% in the derivation cohort vs. 14.76% in our validation cohort). From a clinical and pathological perspective, there were some differences between our validation cohort and the original report’s derivation cohort. These differences may have led to some bias in the subsequent model validation. While both models have some ability to discriminate, starting with a better calibration may have given greater clinical utility to a model that performs a clinical prognostic assessment.

The models in the report were obtained by adjusting data and endpoint events from a clinical prognostic model for adult patients with IgAN constructed by the same team (10, 11). Therefore, this model is deficient in demonstrating ethnicity-recognized risk factors in adults, such as age, MAP, GFR, and proteinuria. A recent review pointed out that the KDIGO guidelines are not well suited for treating children with IgAN, and we need to develop prediction models for the progression of IgAN in children to guide the selection of cases to be treated (18). Therefore, we believe that the selection of risk factors in the original model is biased for pediatric patients.

We first used ROC curves to assess the accuracy of the different risk factors (Supplementary Table S2). The results showed that eGFR had the best accuracy, followed by proteinuria and endocapillary hypercellularity, while the use of immunosuppressive drugs at renal biopsy, segmental glomerulosclerosis, and tubular atrophy/interstitial fibrosis did not have the best predictive accuracy. The data from our medical centre has limitations and a review of the literature was conducted to more accurately evaluate the efficacy of the predictors in the model.

Age at the time of renal biopsy is also a strong considerable risk factor for children with IgAN. Age is recognized as an important risk factor for both adult and pediatric patients (19, 20). For children, with their great developmental potential, different age stages represent different levels of resilience (21). Notably, one large clinical study identified an age of less than 16 years as a protective factor (20).

MAP is a strong risk factor for progression at renal biopsy and follow-up because it is related to proteinuria and decreasing GFR in adults (22). A study showed that the value of MAP at renal biopsy was a significant risk factor for the GFR decline measured by GFR slope in pediatric patients (20).

The GFR at renal biopsy is also a significant predictor of renal function, but its ability to predict outcomes in the pediatric population needs further evaluation. From results observed in the clinic, it is rare to see a decrease in GFR in patients with IgA younger than 18 years old (20). In the originally reported derivation cohort, the authors found a progression of increasing and then decreasing early eGFR in children with IgAN (Supplementary S2). For these reasons, the use of the decreasing GFR as a predictor in younger children may be inaccurate (18).

Proteinuria is a recognized risk factor for clinical progression in the adult population. In contrast, a Japanese study demonstrated no significant correlation between high proteinuria at presentation and a severe prognosis. They also found that children with IgAN had a high rate of spontaneous remission and more sensitivity to corticosteroid treatment (23). These findings may be explained by the fact that, in adults, proteinuria is more likely to indicate chronic renal changes but in children it is likely to indicate proliferative glomerular changes (24). Therefore, high proteinuria at presentation or at renal biopsy is not as convincing as an important prognostic factor in children. This statement is challenged, as some clinical studies have noted that the persistence of high proteinuria during follow-up (time-averaged proteinuria) can be a relevant biomarker in both adults and children (23, 25).

In summary, age and MAP over long-term follow-up still have high predictive efficacy in children. GFR and proteinuria at renal biopsy are less predictive than in adults, but some prospective studies have indicated that trends in GFR and proteinuria over time are better predictors of renal prognosis than their levels at renal biopsy (26).

We also need to focus on histopathology to make a comprehensive prognostic evaluation. A study comparing the differences in histopathology between adults and children with IgAN suggested that glomerular proliferation was associated with urinary protein only in children, and M1, E1, and C1 were the main manifestations in children in the Oxford classification (24). However, histopathology alone does not have predictive value in pediatric patients (20). We need to combine histopathology and clinical findings to achieve significant prediction values (7). Edström Halling S et al. found that E1 and S1 with proteinuria at biopsy appears to be valid predicting renal outcome in children, and Yuko Shima et al. found the same for crescent >30% with proteinuria at biopsy (8, 27). In one study of the VALIGA cohort that compared pathology and outcome in patients with or without glucocorticosteroid/immunosuppressive therapy, 523 patients (46%) received glucocorticoid/immunosuppressive therapy and 622 patients (54%) did not. The results showed that the predictive power of the Oxford classification was reduced by 15% in patients treated with corticosteroids or immunosuppressive agents (28). However, these two drugs are essential in the treatment process for pediatric patients.

For the future construction of predictive models for childhood IgAN, we need to screen for more relevant risk factors based on pediatric data. A Chinese study noted that children with IgAN combined with hypertriglyceridaemia were more likely to have more severe clinical manifestations and pathological changes (29). Thompson A et al. reviewed 13 clinically controlled studies with proteinuria levels as the endpoint event and found that it is reasonable to use a decrease in proteinuria levels to predict IgAN progression (30). Inker LA et al. analysed 11 randomized trials to show that the use of the level of proteinuria as an endpoint for clinical endpoints is reliable (31). Both of these studies shed light on whether proteinuria levels can be used as a study endpoint event in the pediatric population, and we look forward to relevant clinical studies to explore this.

In summary, we have externally validated the full prediction model of IgAN. The validation cohort did not fully align with the derivation cohort in terms of demographic characteristics, clinical baseline levels, treatment, or pathological presentation. These results in the full model with race and the full model without race all did not achieve acceptable performance. We hope that there will be relevant pediatric clinical studies to explore the risk factors for IgAN progression in children and to develop new predictive models for the progression of IgAN in children to guide the selection of treatment.

## Data Availability

The original contributions presented in the study are included in the article/**Supplementary Material**, further inquiries can be directed to the corresponding author.
