# Supplementary material for: Validation of the children international IgA nephropathy prediction tool based on data in Southwest China
Source: Front Pediatr. 2023 Jun 23;11:1183562. doi: 10.3389/fped.2023.1183562 (PMC10327563; doi:10.3389/fped.2023.1183562)
Supplement: Supplementary file 1 [file Datasheet1.docx]

Supplementary Material

Validation of the Children International IgA Nephropathy Prediction Tool Base on Data in Southwest China

**1 Supplementary Data**

The details of the original formula.

**2 Supplementary Figures**

Trends in eGFR in children with IgA nephropathy found in the original article.

**3 Supplementary Tables**

Table 1 The definition of Oxford classification of IgA nephropathy.

Table 2 The predictive accuracy of the predictors in the model.

Supplementary Data: The details of the original formula(1).

The published prediction models for validation were derived as follows:

**The formula for the updated pediatric Prediction Tool model with race/ethnicity**

Predicted risk (time t in months) = 1 - S0(t)Exp[LP]

If t ≤ 36 months, then

LP = 0.156*[sqrt(eGFR)-8.8] + 0.019*(MAP-97) - 0.025*[log(proteinuria) - 0.09] + 0.002*[(MAP* log(proteinuria)) - 8.73] + 0.536*M1 - 0.035*E1 + 0.422*S1 + 0.873*T1 + 0.319*T2 + 0.029*T1*log(proteinuria) - 0.091*T2*log(proteinuria) + 0.055*(age-38) - 0.106*Chinese_race - 1.597*Japanese_race - 0.115*Other_race + 0.066*RASB - 0.060*immunosuppression

If t > 36 months, then

LP = 0.156*[sqrt(eGFR)-8.8] + 0.019*(MAP-97) - 0.025*[log(proteinuria) - 0.09] + 0.002*[(MAP* log(proteinuria)) - 8.73] + 0.536*M1 - 0.035*E1 + 0.422*S1 + 0.873*T1 + 0.319*T2 + 0.029*T1*log(proteinuria) - 0.091*T2*log(proteinuria) + 0.055*(age-38) + 0.219*Chinese_race - 1.597*Japanese_race - 0.115*Other_race + 0.066*RASB - 0.060*immunosuppression

S0(t) = 1.02701 - 0.319477*[(t+0.1)/100]0.5 - 0.083333*[(t+0.1)/100]2

**The formula for updated pediatric Prediction Tool model without race/ethnicity**

Predicted risk (time t in months) = 1 - S0(t)Exp[LP]

LP = 0.104*[sqrt(eGFR)-8.8] + 0.027*(MAP-97) - 0.012*[log(proteinuria) - 0.09] + 0.001*[(MAP* log(proteinuria)) - 8.73] + 0.663*M1 - 0.012*E1 + 0.381*S1 + 0.238*T1 + 0.420*T2 + 0.034*T1*log(proteinuria) - 0.109*T2*log(proteinuria) +0.043*(age-38) + 0.040*RASB + 0.056*RASB* log(proteinuria) - 0.090*immunosuppression

S0(t) = 1.01792 - 0.220482*[(t+0.1)/100]0.5 - 0.09333*[(t+0.1)/100]2

where the log is the natural log function.

Supplementary Figures: Trends in eGFR in children with IgA nephropathy found in the original article(1).

A comparison of the eGFR change curves shows that the decline in eGFR in pediatric patients after the age of 15-18 years is similar to that of adults. The developmental trajectory of IgAN in children is non-linear.


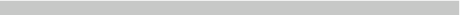

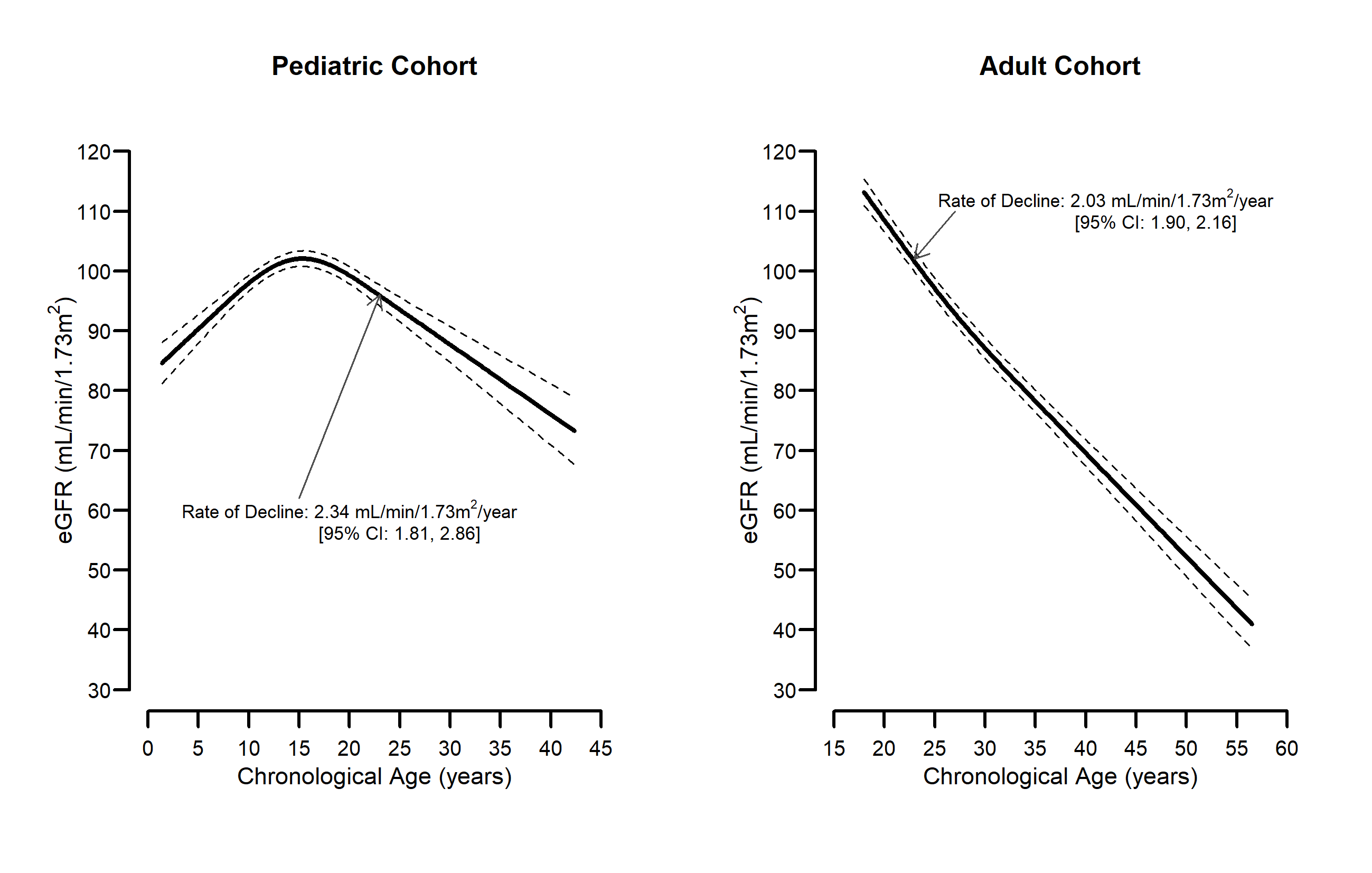


Supplementary Tables:

Table 1 The definition of Oxford classification of IgA nephropathy(2).

| Histological variable | Definition | Score |
| --- | --- | --- |
| Mesangial hypercellularity | More than four mesangial cells in any mesangial area of a glomerulus | M0: <50% of glomeruli showing mesangial hypercellularity  M1: >50% of glomeruli showing mesangial hypercellularity |
| Endocapillary hypercellularity | Hypercellularity due to an increased number of cells within glomerular capillary lumina | E0: no endocapillary hypercellularity  E1: any glomeruli showing endocapillary hypercellularity |
| Segmental glomerulosclerosis | Adhesion or sclerosis (obliteration of capillary lumina by matrix) in part but not the whole glomerular tuft | S0: absent  S1: present in any glomeruli |
| Tubular atrophy/interstitial fibrosis | Estimated percentage of cortical area showing tubular atrophy or interstitial fibrosis, whichever is greater | T0: 0-25% of cortical area  T1: 26-50% of cortical area  T2: >50% of cortical area |

Table 2 The predictive accuracy of the predictors in the model.

| Risk factor | AUC (95% CI) | Risk factor | AUC (95% CI) |
| --- | --- | --- | --- |
| Age | 0.535 (0.398-0.672) | Standardized MAP | 0.561 (0.460-0.633) |
| eGFR at biopsy | 0.905 (0.842-0.969) | Proteinuria | 0.623 (0.502-0.744) |
| M | 0.528 (0.401-0.656) | E | 0.618 (0.502-0.733) |
| S | 0.534 (0.407-0.661) | T | 0.530 (0.403-0.656) |
| use of RASB | 0.545 (0.417-0.675) | use of immunosuppression | 0.503 (0.380-0.625) |

1. Barbour SJ, Coppo R, Er L, Russo ML, Liu ZH, Ding J, et al. Updating the International IgA Nephropathy Prediction Tool for use in children. Kidney Int. 2021;99(6):1439-50.

2. Markowitz G. Glomerular disease: Updated Oxford Classification of IgA nephropathy: a new MEST-C score. Nat Rev Nephrol. 2017;13(7):385-6.
